# A Unique Spumavirus Gag N-terminal Domain with Functional Properties of Orthoretroviral Matrix and Capsid

**DOI:** 10.1371/journal.ppat.1003376

**Published:** 2013-05-09

**Authors:** David C. Goldstone, Thomas G. Flower, Neil J. Ball, Marta Sanz-Ramos, Melvyn W. Yap, Roksana W. Ogrodowicz, Nicole Stanke, Juliane Reh, Dirk Lindemann, Jonathan P. Stoye, Ian A. Taylor

**Affiliations:** 1 Division of Molecular Structure, MRC National Institute for Medical Research, the Ridgeway, Mill Hill, London, United Kingdom; 2 Division of Virology, MRC National Institute for Medical Research, the Ridgeway, Mill Hill, London, United Kingdom; 3 Institute of Virology, Technische Universität Dresden, Dresden, Germany; Vanderbilt University School of Medicine, United States of America

## Abstract

The *Spumaretrovirinae*, or foamyviruses (FVs) are complex retroviruses that infect many species of monkey and ape. Although FV infection is apparently benign, trans-species zoonosis is commonplace and has resulted in the isolation of the Prototypic Foamy Virus (PFV) from human sources and the potential for germ-line transmission. Despite little sequence homology, FV and orthoretroviral Gag proteins perform equivalent functions, including genome packaging, virion assembly, trafficking and membrane targeting. In addition, PFV Gag interacts with the FV Envelope (Env) protein to facilitate budding of infectious particles. Presently, there is a paucity of structural information with regards FVs and it is unclear how disparate FV and orthoretroviral Gag molecules share the same function. Therefore, in order to probe the functional overlap of FV and orthoretroviral Gag and learn more about FV egress and replication we have undertaken a structural, biophysical and virological study of PFV-Gag. We present the crystal structure of a dimeric amino terminal domain from PFV, Gag-NtD, both free and in complex with the leader peptide of PFV Env. The structure comprises a head domain together with a coiled coil that forms the dimer interface and despite the shared function it is entirely unrelated to either the capsid or matrix of Gag from other retroviruses. Furthermore, we present structural, biochemical and virological data that reveal the molecular details of the essential Gag-Env interaction and in addition we also examine the specificity of Trim5α restriction of PFV. These data provide the first information with regards to FV structural proteins and suggest a model for convergent evolution of *gag* genes where structurally unrelated molecules have become functionally equivalent.

## Author Summary

Foamyviruses (FVs) or spuma-retroviruses derive their name from the cytopathic effects they cause in cell culture. By contrast, infection in humans is benign and FVs have entered the human population through zoonosis from apes resulting in the emergence of Prototypic Foamyvirus (PFV). Like all retroviruses FVs contain *gag*, *pol* and *env* structural genes and replicate through reverse-transcription and host genome integration. Gag, the major structural protein, is required for genome packaging, virion assembly, trafficking and egress. However, although functionally equivalent, FV and orthoretroviral Gag share little sequence homology and it is unclear how they perform the same function. Therefore, to understand more about the relationship between FV and orthoretroviral replication we have carried out structural/virological studies of PFV Gag. We present the structure of Gag-NtD, a unique domain found only in FV Gag and show that despite functional equivalence, Gag-NtD is entirely structurally unrelated to orthoretroviral Gag. We also provide the molecular details of an essential interaction between Gag-NtD and the FV Envelope and demonstrate that Gag-NtD contains the determinants of Trim5α restriction. Our findings are discussed in terms of evolutionary convergence of retroviruses and the implications of alternative arrangements of Gag on pattern recognition by viral restriction factors.

## Introduction

Spuma- or foamy viruses (FVs) are complex retroviruses and constitute the only members of the *Spumaretrovirinae* subfamily within the *Retroviridae* family. They have been isolated from a variety of primate hosts [1], [2], [3], [4] as well as from cats [5], [6], [7], cattle [8], horses [9] and sheep [10]. Endogenous FVs have also been described in sloth [11], aye-aye [12] and coelacanth [13]. Prototypic foamy virus (PFV) is a FV isolated from human sources [14], [15]. The PFV genome is highly similar to that of isolates of simian foamy virus from chimpanzee (SFV_cpz_) and so infection in humans is believed to have arisen through a zoonotic transmission [16], [17], [18]. Nevertheless, even though FVs are endemic within non-human primates and display a broad host range, human-to-human transmission of PFV has never been detected. Moreover, although in cell culture FV infection causes pronounced cytopathic effects [19] infection in humans is apparently asymptomatic [20], [21], [22] making their usage as vectors for gene therapy an attractive proposition [23].

FVs share many similarities with other retroviruses in respect of their genome organisation and life cycle. However, they vary from the *Orthoretrovirinae* in a number of important ways. These include the timing of reverse transcription that occurs in virus producer cells rather than newly infected cells [24], [25] and the absence of a Gag-Pol fusion protein [26] [27]. In addition, the Gag protein remains largely unprocessed in FVs [28] whereas within the *Orthoretrovirinae* processing of the Gag polyprotein represents a critical step in viral maturation, producing the internal structural proteins Matrix (MA), Capsid (CA) and Nucleocapsid (NC) found in mature virions. Furthermore, FV Gag lacks the Major Homology Region (MHR) and Cys-His boxes found in orthoretroviral CA and NC, respectively.

Also unique to FVs is a requirement for the interaction of the Gag protein with the viral envelope protein (Env) in order to bud from the producer cell [29], [30], [31]. Nevertheless, despite these profound dissimilarities, the Gag protein contains the cytoplasmic targeting and retention signal (CTRS) [32], [33], [34], essential for both FV and betaretrovirus replication. Moreover, in all retroviral subfamilies Gag carries out the same functional roles including assembly, nucleic acid packaging, transport to and budding through the cytoplasmic membrane of the producer cell as well as trafficking through the cytoplasm of the target cell and uncoating. Similarly, FV Gag also contains the determinants for restriction by Trim5α [35], [36] that in orthoretroviruses are the residues displayed on the assembled CA lattice [37].

To date, high resolution X-ray and/or NMR structures have been reported for MA, CA and NC components of Gag from numerous retroviruses [38], [39], [40], [41], [42], [43], [44], [45], [46], [47]. However, structural information with regard to the Gag of FVs has remained elusive and is vital for any detailed understanding of how FV Gag fulfils its many functions. Here we report the crystal structure an amino terminal domain from the Gag of PFV (PFV-Gag-NtD), provide the molecular details of the interaction of this domain with the N-terminal leader sequence from the PFV Envelope (PFV-Env) and demonstrate that the PFV-Gag-NtD is also the target for Trim5α restriction factors. Our data reveal that the FV Gag is unique and structurally unrelated to the Gag protein of other retroviruses. Nevertheless, the Gag-NtD has functional properties associated with both the MA and CA proteins of the orthoretroviruses. These findings have important implications for the evolution of FVs and the mechanism of virus restriction by Trim5α.

## Results

### Structure of the PFV-Gag-NtD

**Figure 1:**
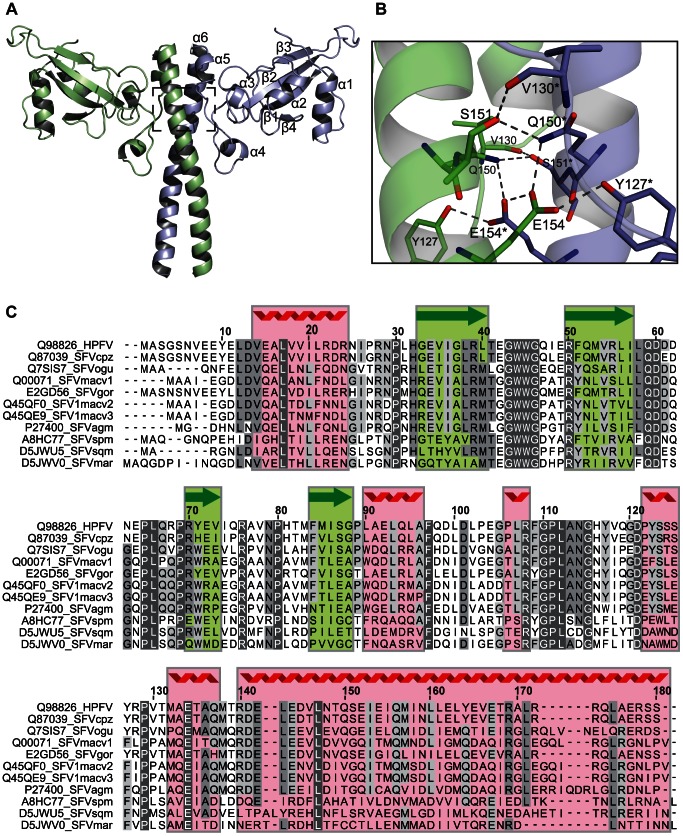
The crystal structure of the amino-terminal domain of PFV Gag. (**A**) Cartoon representation of the PFV Gag-NtD homodimer, Monomer-A is shown in pale blue and Monomer-B in green. The secondary structure elements are numbered sequentially from the amino-terminus on Monomer-A. (**B**) Details of the Gag-NtD dimer interface located at the centre of the coiled-coil region and boxed in **A**. Residues that contribute to the intermolecular hydrogen-bonding network are labelled and shown in stick representation. Those with asterisks are from Monomer-A. Intermolecular hydrogen bonding interactions are shown as dashed lines. (**C**) Sequence alignment of foamyvirus Gag-NtDs from apes, old and new world monkeys, numbering corresponds to the PFV sequence. The position of secondary structure elements in PFV is indicated above the sequence, red coil for helices and green arrows for strands. Regions with greatest sequence homology are highlighted with grey boxes. Residues that are conserved in all sequences are also coloured white. Sequences are annotated with the database accession number and species are abbreviated as follows cpz, Chimpanzee; ogu, Orangutan; mac, Macaque; gor, Gorilla; agm, African green monkey; spm, Spider monkey; sqm, Squirrel monkey; mar, marmoset.

PFV Gag is a 648 polypeptide and the major FV structural protein in the assembled virion. Bioinformatic analysis of the primary sequence and that of related FVs suggested that the N-terminal 179 residues of PFV-Gag comprised a stable domain (PFV-Gag-NtD). This fragment was expressed in *E. coli* and subsequently the crystal structure determined by SAD methods and refined at a resolution of 2.4 Å. The final R_work_/R_free_ are 17.2% and 23.0% respectively. Details of the structure solution and refinement are presented in **Table 1**. In the crystal, the asymmetric unit comprises a dimer of the protein with residues 9–179 clearly visible in the electron density map for both monomers. The residues preceding Glu9 along with the N-terminal His-tag are not visible and presumably disordered in the crystal. The structure of PFV-Gag-NtD dimer, **Figure 1A**, comprises a mixed alpha-beta fold dominated by a large central coiled-coil, resembling a two-bladed propeller. The N-terminal region of the protein forms a head domain containing a central 4-stranded β-sheet together with two helices, α1 and α2, that pack against one side of the sheet forming a tight hydrophobic core. The loop between strands β3 and β4 crosses to the opposing side of the sheet where helix α2 leads into a region lacking secondary structure that precedes three further short helices α3, α4 and α5. Helix α5 is immediately followed by α6, a long helix (58 Å) comprising residues Arg140-Ser179 that forms the coiled coil domain making the majority of interactions between the two monomers. The observation of this unusual arrangement prompted us to examine the structural relatedness of the PFV-Gag-NtD with the Gag-derived proteins from other retroviruses and those of hepadnavirus. However, similarity searches undertaken using the DALI [48] and SSM [49] search engines revealed no significant homology between the PFV-Gag-NtD and the retroviral MA or CA. In fact, no homology was detected with any structure deposited in the PDB making the foamy virus Gag-NtD at present unique.

**Table 1:** X-ray data collection and structure refinement.

Data collection	HFV Gag 1–179 (NTD)	HFV Gag-NTD/Env
Space group	P2_1_	P2_1_
Cell dimensions		
a, b, c (Å)	35.4, 94.7, 61.7	59.4, 122.4, 61.8
α, β, γ (°)	90, 93.7, 90	90, 97.5, 90
	*peak*	
Wavelength (Å)	0.9791	0.9763
Resolution (Å)	25-2.4 (2.49-2.4)	45.5-2.9 (3.0-2.9)
*R*_sym_ or *R*_merge_	6.1 (11.0)	7.7 (38.1)
I/σI	17.4 (9.2)	14.6 (4.3)
Completeness (%)	98.1 (95.8)	99.3 (99.6)
Redundancy	3.2 (2.8)	3.3 (3.5)
**Refinement**		
Resolution (Å)	25-2.4	45.5-2.9
No. reflections		19332
*R*_work_/*R*_free_	17.2/23.0	22.6/27.1
No. atoms		
Total	2985	5931
Protein	2814 (344)	5922
Water	171	9
*B*-factors		
Overall	23.0	47.8
Protein	22.9	47.9
Water	25.2	24.6
R.m.s. deviations		
Bond lengths (Å)	0.008	0.005
Bond angles (°)	1.043	0.839

### Dimer interface

The PFV-Gag-NtD dimer interface buries approximately 1700 Å^2^ of the monomer surface area. The large central coiled-coil formed by helix α6 comprises the majority of this interface, supplemented by residues in helices α4 and α5 and the adjoining loop. The coiled-coil contains three regions of leucine zipper at residues Leu143/147, Leu160/161 and Leu171/175. Additionally, a highly synergistic hydrogen-bonding network centred on residue Glu154 is located between two of the zipper regions. Here, the Glu154 sidechain forms hydrogen bonds with the sidechains of Glu154*, Gln150* and Tyr127* of the opposing monomer. Gln150 makes further hydrogen bonds with Ser151* that in turn is hydrogen bonded to the mainchain carbonyl of Val130, **Figure 1B**. The loop between helix α4 and α5 runs alongside this region also making several interactions. In addition, at the amino terminus of the coiled-coil, Arg140 makes bifurcated hydrogen bonds with the backbone hydroxyl of Met138 and the sidechain hydroxyl of Glu144 as well as further hydrogen bonds with the backbone of Ala136 in helix α4 and the side chain of Asp141* in the opposing monomer. This extensive network of intermolecular protein-protein interactions and large molecular interface of 1700 Å^2^ is nearly twice that of the HIV-1 CA-CtD dimer interface, 920 Å^2^ [50], [51] and suggests that Gag-NtD of PFV forms a tightly associated dimer. Moreover, sequence alignment with the N-terminal region of Gag from other primate foamy viruses, **Figure 1C**, reveals strong sequence conservation in loops and secondary structure elements in the head domains together with several buried hydrophobic residues in the coiled coil indicating that a conserved dimeric Gag N-terminal domain is likely a feature of the primate foamy viruses.

### Solution conformation of Foamyvirus Gag-NtDs

**Figure 2:**
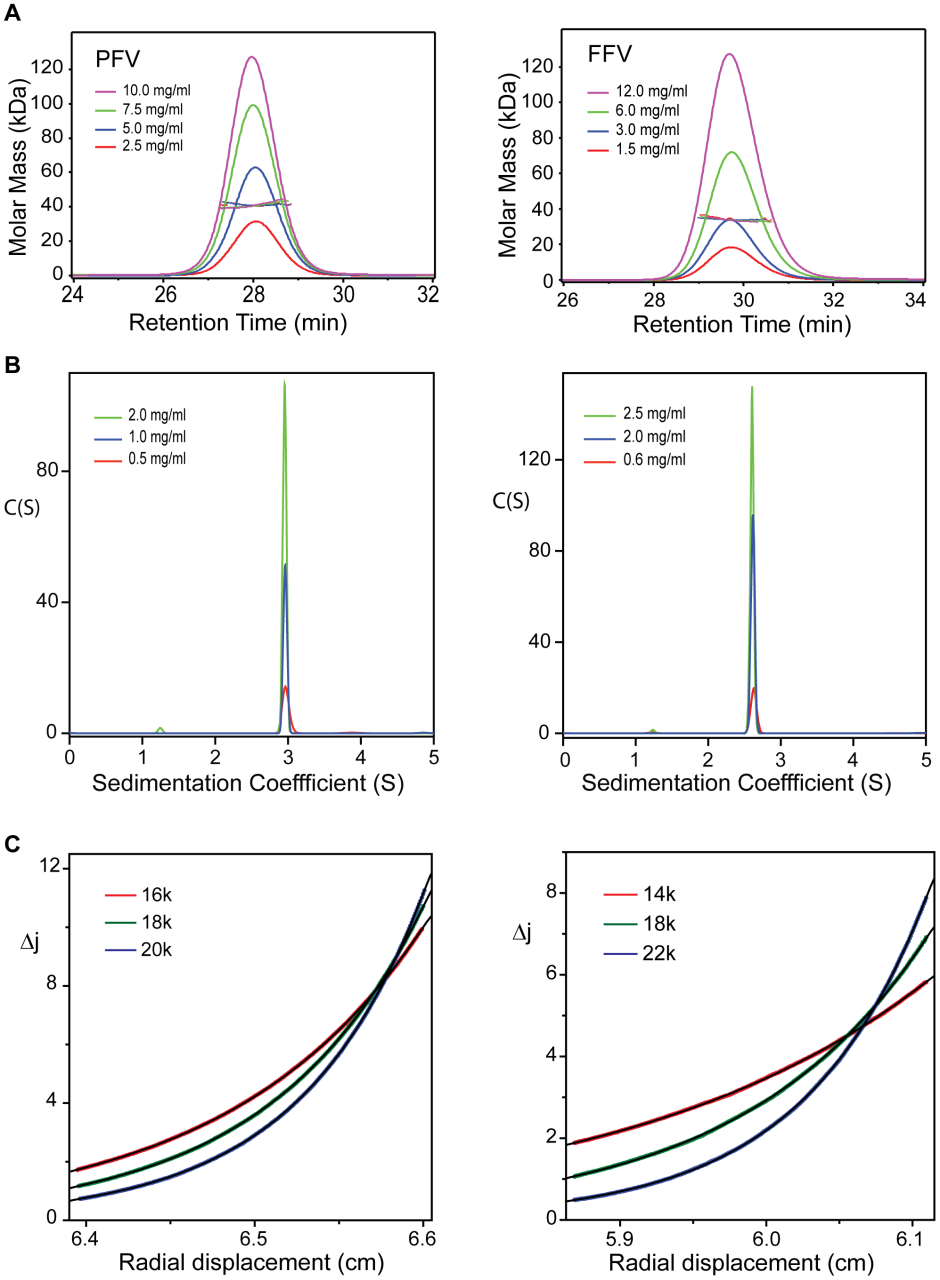
Conformation and solution oligomeric state of FV-Gag-NtDs. (**A**) SEC-MALLS analysis of PFV-Gag-NtD recorded at 2.5 mgml^−1^ (red), 5.0 mgml^−1^ (blue), 7.5 mgml^−1^ (green) and 10.0 mgml^−1^ (pink) (left panel) and FFV Gag NtD at 1.5 mgml^−1^ (red) 3.0 mgml^−1^ (blue), 6.0 mgml^−1^ (green) and 12.5 mgml^−1^ (pink) (Right panel). Differential refractive index (dRI) is plotted against retention time and the molar mass distributions, determined throughout the elution of each peak, plotted as points. (**B**) C(S) distributions derived from sedimentation velocity data recorded from PFV-Gag-NtD at 0.5 mgml^−1^ (red), 1.0 mgml^−1^ (blue) and 2.0 mgml^−1^ (green) (left panel) and FFV-Gag-NtD at 0.6 mgml^−1^ (red), 2.0 mgml^−1^ (blue) and 2.5 mgml^−1^ (green) (right panel). (**C**) Multi-speed sedimentation equilibrium profiles determined from interference data collected on PFV-Gag-NtD at 67 µM (left panel) and FFV-Gag-NtD at 50 µM (right panel). Data was recorded at the three speeds indicated. The solid lines represent the best fit to the data using a single species model.

Given the unexpected nature of the dimer observed in the crystal structure, the conformation and self-association properties of the Gag-NtD from PFV and from the non-primate feline foamy virus (FFV) were examined using a variety of solution hydrodynamic methods. Initial assessment by Size Exclusion Chromatography coupled Multi-Angle Laser Light Scattering (SEC-MALLS), over a range of protein concentration (12-1.5 mg/ml), yielded invariant solution molecular weights of 40.0 kDa and 34.0 kDa for PFV- and FFV-Gag-NtD respectively, **Figure 2A**. By comparison, the sequence-derived molecular weights are 22.8 kDa and 19.0 kDa. Given these values together with the lack of a concentration dependency of the molecular weight it is apparent that along with PFV, the Gag-NtD from FFV also forms strong dimers in solution. To confirm the oligomeric state, velocity (SV-AUC) and equilibrium (SE-AUC) analytical ultracentrifugation of PFV- and FFV-Gag-NtD was undertaken. A summary of the experimental parameters, molecular weights derived from the data and statistics relating to the quality of fits are shown in **Table 2**. Analysis of the sedimentation velocity data for PFV-Gag-NtD revealed no concentration dependency of the sedimentation coefficient (S_20,w = _3.08) over the range measured, **Figure 2B**. Similar data were obtained for FFV-Gag-NtD (S_20,w = _2.72) indicating both proteins are single stable species. The molecular weights derived from either C(S) or discrete component analysis were 47 kD and 36 kD respectively, **Table 2**, consistent with a PFV- and FFV-Gag-NtD dimer. The frictional ratios (*f*/*f_o_*) obtained from the analysis, 1.4–1.5, also suggest both dimers have a similar elongated conformation. Multispeed sedimentation equilibrium studies at varying initial protein concentration were also carried out and typical equilibrium distributions for PFV- and FFV-Gag-NtD from individual multispeed experiments are presented in **Figure 2C**. Analysis of individual gradient profiles showed no concentration dependency of the molecular weight and so data were fit globally with a single ideal molecular species model, producing weight averaged molecular weights of 44 kDa and 33.7 kDa for PFV- and FFV-Gag-NtD respectively. These data confirm that formation of stable dimeric structures is a common property shared among the Gag proteins of divergent FVs and N-terminal domain mediated dimerisation is likely an important component of FV assembly.

**Table 2:** Hydrodynamic parameters of PFV- and FFV-Gag-NtD.

Protein	HFV ht-(M1-S179)	FFV ht-(M1-V154)
**Parameter**		
ν (ml.g^−1^)	0.731	0.732
ρ (g.ml^−1^)	1.005	1.005
aM_r_	22,713	18,999 (17,146)
ε_280_ (M^−1^ cm^−1^)	19,200	15,300
**Sed velocity**		
C_range_ (µM)	20–90	30–130
S_20,w_ (×10^13^) sec	3.08±0.02	2.72±0.01
D_20,w_ (×10^7^) cm^2^ sec^−1^	5.96±0.04	6.81±0.3
bM_w_ (S/D) kD	46.7±0.2	36.1±1.5
cƒ/ƒ_0_ (S_20,w_)	1.48	1.49
dƒ/ƒ_0_ (D_20,w_)	1.52	1.42
M_w_ C(S) kD	48.2±1	37.4±1
eƒ/ƒ_0_ C(S)	1.54	1.47
frmsd C(S)	0.004–0.008	0.003–0.011
**Sed eqm**		
C_range_ (µM)	30–100	14–50
gM_w_ kD	44±2	h33.7±1
irmsd	0.003–0.005	0.004–0.006

a Molar mass calculated from the protein sequence value in parenthesis is after removal of the N-terminal His-tag.

b The weight averaged molecular weight from discrete component analysis.

c The frictional ratio calculated from S_20,w_ from discrete component analysis.

d The frictional ratio calculated from D_20,w_ from discrete component analysis.

e The weight-averaged frictional ratio from the best fit C(S) distribution function.

f The range of the rms deviations observed when data were fitted using a continuous sedimentation coefficient distribution model.

g The weight averaged molecular weight from Global SE analysis.

h Molecular weight derived from a FFV-154 sample with the His tag removed.

i The range of the rms deviations observed for each multi-speed sample when fitted individually.

### The Gag-NtD-Env interaction

**Figure 3:**
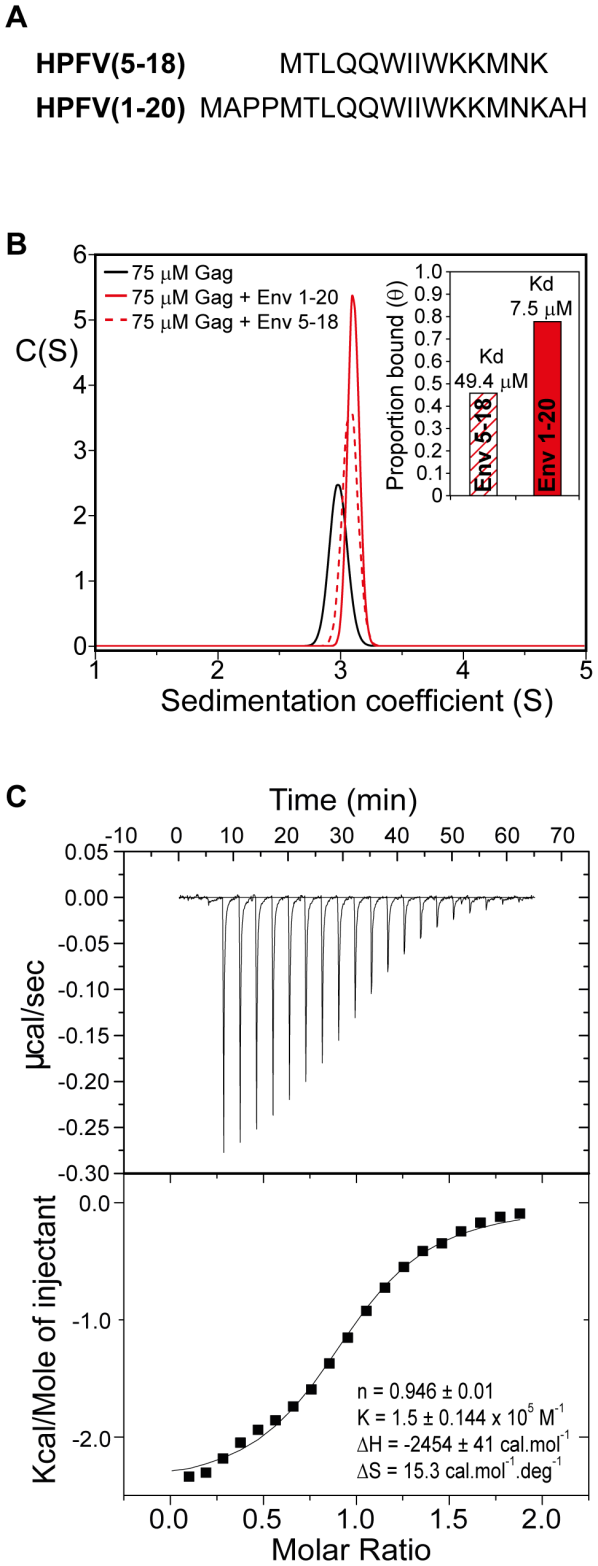
Analysis of PFV Gag-Env interactions. (**A**) Peptide sequences from the PFV Env leader used in binding experiments. (**B**) Sedimentation coefficient distribution functions, C(S) that best fit sedimentation velocity profiles from PFV-Gag-NtD (black) and from 75 µM equimolar mixtures of PFV-Gag-NtD with Env_1–20_ (solid red) and Env_5–18_ (dashed red). Inset, proportion bound quantified as described in methods and equilibrium dissociation constants derived from these data. (**C**) Interaction of PFV-Gag-NtD with Env_1–20_ quantified by ITC. The top panel shows the raw thermogram and the bottom panel shows the titration data along with best line of best fit and the fitted parameters (inset).

The interaction of foamy virus Gag and Env proteins is a requirement for successful budding and the production of infectious particles [52]. Mutations in either Gag-NtD or the N-terminal leader peptide region of Env (Env-LP) have been shown to block viral egress [31], [53], [54]. To better understand this interaction and shed light on how FV Gags recruit Env, we examined the interaction of the PFV-Gag-NtD with the PFV-Env-LP using SV-AUC. Sedimentation data were recorded for Gag-NtD and for equimolar mixtures of Gag-NtD with either of two Env leader peptides, residues 5–18 or 1–20, **Figure 3A**. The data were fitted using the C(S) distribution of sedimentation coefficients and the integrated absorbance of the fast moving Gag-NtD 3S component then quantified. In samples containing peptide-protein mixtures a small increase in the apparent sedimentation coefficient of the 3S boundary is apparent, accompanied by an increase in the integrated absorbance, **Figure 3B**. This shift and absorbance increase results from association of the strongly absorbing Env peptides with the PFV-Gag-NtD (ε_280_ = 11,400 M^−1^ cm^−1^) and simple quantitation of the absorbance change reports the proportion of peptide bound and association constant for the interaction (see methods). In this way an equilibrium association constant of 2.0×10^4^ M^−1^ for the Gag-NtD interaction with Env residues 5–18 (Env_5–18_) and 1.3×10^5^ M^−1^ for the interaction with Env residues 1–20 (Env_1–20_) was determined. To confirm this observation the interaction of Env_1–20_ with PFV-Gag-NtD was examined using isothermal titration calorimetry (ITC). The results presented in **Figure 3C** reveal a 1∶1 stoichiometry where each monomer of the PFV-Gag-NtD binds a single Env peptide with an equilibrium association constant of 1.5×10^5^ M^−1^ consistent with the SV-AUC experiments.

### Structure of the PFV-Gag-NtD-Env complex

**Figure 4:**
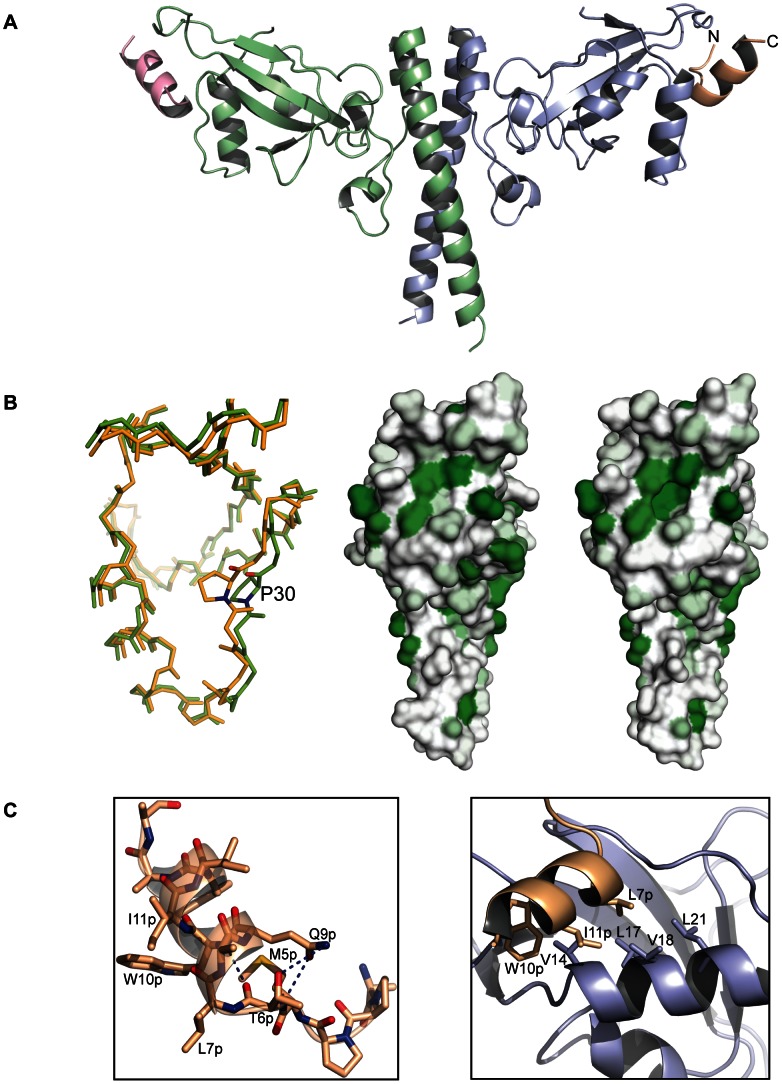
Structure of the PFV Gag-Env complex. (**A**) Cartoon representation of the Gag-Env complex. The Gag-NtD homodimer is shown in the same orientation and colour scheme as in **Figure 1**. The helical Env peptides bound at the periphery of each head domain are coloured magenta and gold with N- and C-termini indicated. (**B**) A structural alignment shown in stick representation of free (orange) and bound (green) Gag-NtD is shown in the left-hand panel. The view is looking into the Env binding site at 90-degree rotation from that in **A**. Residue P30 is highlighted to show the backbone movement that occurs in the α1-β1 loop upon Env binding. The central and right-hand panels show the distribution of surface hydrophobicity on the Gag-NtD in the free and bound structures respectively. Hydrophobicity is represented by green shading with darker regions representing the most hydrophobic areas. The backbone movement of the α1-β1 loop in the bound structure (right panel) opens up a hydrophobic pocket in order to accommodate the Env peptide. (**C**) A cartoon representation of the bound PFV Env_1–20_ peptide is shown in the left hand panel. Intramolecular hydrogen bonding between residues in the N-terminal extended region and those in the helical section are displayed as dashed lines. Residues with apolar and aromatic side chains that line one face of the helix are also labelled. Details of the Gag-Env interface are shown in the right hand panel. Gag and Env molecules are coloured as in **A**. Residues with apolar side chains that contribute to the hydrophobic interface are shown in stick representation.

The structure of the PFV-Gag-NtD bound to the Env_1–20_ leader peptide was determined by molecular replacement and refined at a resolution of 2.9 Å with a final R_work_/R_free_ of 22.6% and 27.1% respectively, **Table 1**. The asymmetric unit comprises two dimers of the complex with residues 9–179 of the Gag-NtD clearly visible in the electron density map for two of the four monomers and residues 9–170 in the two remaining protomers. Four helical Env peptides are also present, bound at the periphery of each head domain close to α1 and the associated α1-β1 loop of the Gag-NtD monomers, **Figure 4A**. Largely, the conformation of the Gag-NtD head and stalk domains are the same as in the free structure (RMSD of 0.4 Å between all equivalent Cα atoms) excepting some small differences in the conformation of the β3–β4 loop, **Supplementary Figure S1**. However, in the bound structure the α1-β1 loop around the highly conserved residue Pro30 undergoes a concerted 2.5 Å shift, **Figure 4B** and **Supplementary Figure S1**. Comparison of surface hydrophobicity profiles of the free and bound structures **Figure 4B**, reveals that this movement opens the Env binding site exposing a deep apolar pocket to accommodate the hydrophobic side chains from the Env peptide. In the complex residues Met1^Env^ to Thr6^Env^ of Env constitute an extended N-terminal region and Leu7^Env^ to Met16^Env^ form the hydrophobic α-helix bound to Gag. Hydrogen bonding between the sidechains of Thr6^Env^ in the N-terminal region and Gln9^Env^ on the amino-terminal turn of the Env helix provides stabilising interactions that maintain the helical conformation of the Env, **Figure 4C**. Inspection of Gag-Env interface reveals a network of hydrophobic interactions with the apolar and aromatic sidechains of Leu7^Env^, Trp10^Env^ and Trp13^Env^ on one face of the Env helix packing against the Val14, Leu17, Val18 and Leu21 sidechains on α1 of Gag, **Figure 4C**. In particular, the side chain of Leu7^Env^ is seated in the apolar pocket in the Gag-NtD were it makes hydrophobic interactions with the aliphatic side chains of both Leu17 and Leu21. Val14 packs against the ring of Trp10^Env^ that also makes a hydrogen bonding interaction between the indole Nε proton and the carbonyl of Leu66 in the β2–β3 loop. This hydrophobic interface is accompanied by a number of polar contacts between the backbone of residues Ala2^Env^, Pro3^Env^ and Met5^Env^ in the Env N-terminal extended region with the sidechains of Asn63 and Gln59 in the β2–β3 loop and the mainchain of His32 and Pro30 in the α1-β1 loop. The bound conformation is further stabilised by an accompanying helix capping interaction between the Asn29 sidechain and the N-terminal turn of the Env helix.

**Figure 5:**
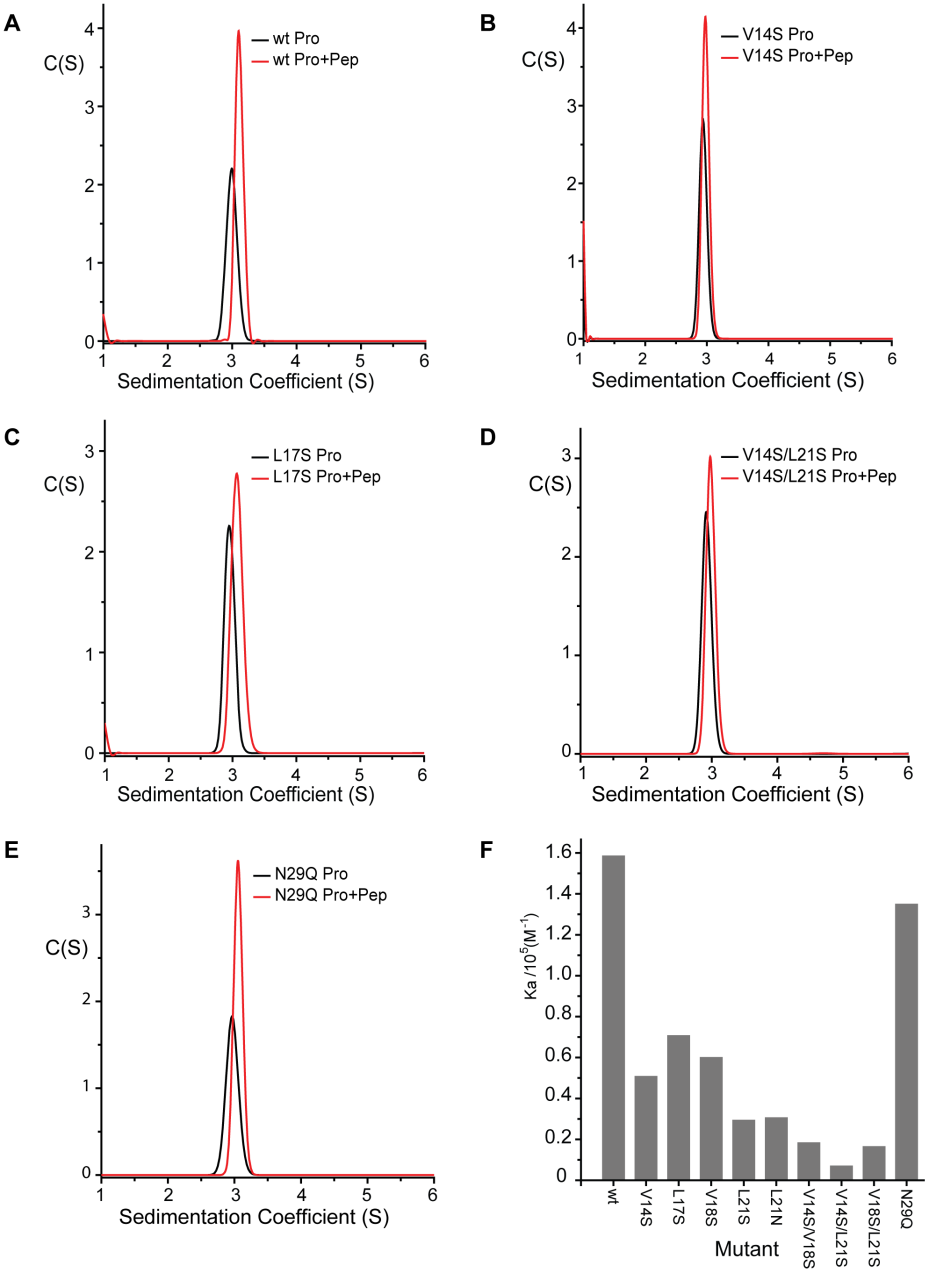
Sedimentation velocity analysis of Gag-Env interface mutants. C(S) functions that best fit sedimentation velocity profiles from (**A**) *wt* PFV-Gag-NtD, (**B**) V14S, (**C**) L17S, (**D**) V14S/L21S and (**E**) N29Q mutants. The C(S) function from 75 µM Gag-NtD (black) and from 75 µM equimolar mixtures of Gag-NtDs with Env(1–20) (red) are shown in each panel. (**F**) Histogram of equilibrium association constants derived from the sedimentation data as described in methods.

In order to probe the importance of the interactions in the Gag-Env interface observed in the crystal structure a series of serine and asparagine substitutions were introduced at Val14, Leu17, Val18 and Leu21 to make the Env binding site progressively polar. In addition, in order to examine the contribution of the α1-β1 loop to the Env interaction a conservative Asn29 to Gln substitution was also introduced. The affinity of binding of these Gag-NtD mutants to Env-LP was examined using the sedimentation velocity assay, **Figure 5A–E** and **Supplementary Figure S2**. In all cases, the single polar substitutions introduced into the Env binding site reduced the affinity of the Gag-Env interaction. The decrease varied from 5 – 2 fold in the order Leu21>Val14>Leu17≈Val18 identifying these residues as being required for the Gag-Env interaction. Double substitutions decreased the affinity even further with the Val14/Leu21 to serine having the greatest effect, resulting in around a twenty-fold reduction in binding, **Figure 5F**. Moreover, the triple substitution where Val14, Val18 and Leu21 were all substituted by serine reduced binding to an undetectable level, **Supplementary Figure S2**. The conservative change Asn29 to glutamine has little effect on Env binding perhaps reflecting the importance of the backbone movement around Pro30 rather than sidechain interactions for Env-binding at this position.

### Budding and infectivity

**Figure 6:**
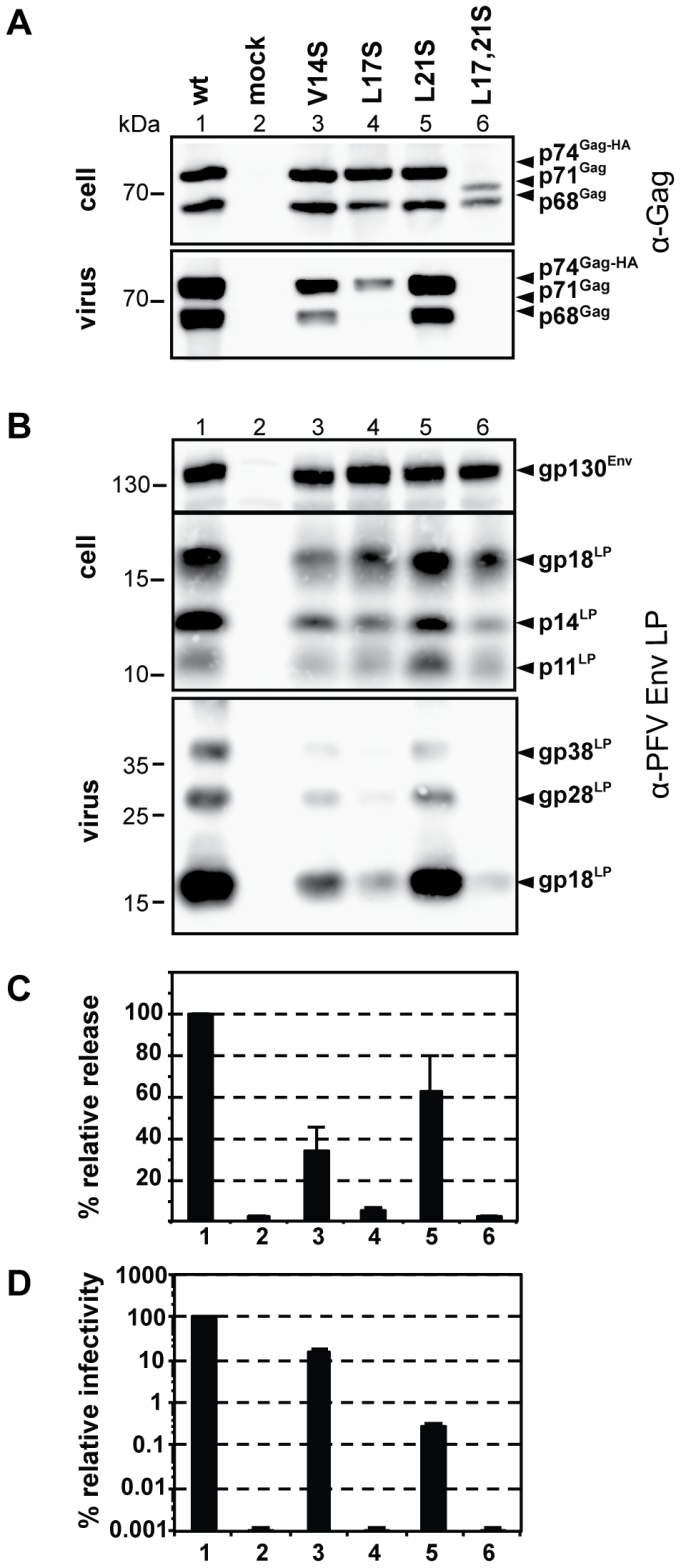
Infectivity and particle budding of Gag mutants. 293T cells were co-transfected with equal amounts of PFV transfer vector puc2MD9, Env packaging construct pcoPE, Pol packaging construct pcoPP and various Gag packaging constructs (*wt*: pcziPG CLHH; V14S: pcziPG CLHH V14S; L17S: pcziPG CLHH L17S; L21S: pcziPG CLHH L21S; L17, 21S: pcziGag4 L17, 21S) or only with pUC19 (mock) as indicated. Western blot analysis of cell lysates (cell) and pelleted viral supernatants (virus) using (**A**) polyclonal antibodies specific for PFV-Gag (α-Gag) or (**B**) rabbit polyclonal antibodies specific for PFV Env-LP (α-LP). The identity of the individual proteins is indicated on the right. (**C**) Relative amounts of released Gag and RT quantified from Western blots from two independent experiments (n = 2–4). (**D**) Relative infectivity of extracellular 293T cell culture supernatants using an eGFP marker gene transfer assay were determined 3 days post infection. The values obtained using the wild type Gag packaging vector were arbitrarily set to 100%. Absolute titres of these plain supernatants were 8.7±3.3×10^6^ EGFP ffu/ml. Means and standard deviations of three independent experiments (n = 3–6) are shown.

It has been shown previously that mutation of Leu17 in PFV-Gag-NtD gives rise to viral defects and negatively affects viral egress. Substitution by alanine has only minor effects on Env incorporation and particle release but progeny particles show a severe reduction of the infectivity. In contrast, serine substitution results in a loss of viral budding capacity [53]. To assess *in vivo* effects of serine substitution at Leu17 and at other positions in the Gag-Env interface the Leu17, Val14 and Leu21 to Ser mutations that disrupt the Gag-Env interaction *in vitro* were introduced and transfected cells assayed for particle production as well as Env/Gag incorporation and viral infectivity, **Figure 6**. In these *in vivo* experiments, the greatest effects were seen with Leu17 and Leu17/Leu21 mutant viruses that show greatly reduced levels of Gag released into the supernatant compared to wild type. By contrast, only a small reduction in Gag release was observed in the Val14 virus and in the Leu21 virus the amount of Gag is comparable to *wt*, **Figure 6A**. Examination of Env production and processing in the producer cells reveals it is unaffected by any of the mutations, **Figure 6B**. However, Env incorporation into virions is greatly reduced in both the Leu17 and Leu17/Leu21 particles, moderately reduced in the Val14 virus and that near *wt* levels are present in Leu21 particles. These results are mirrored when particle release was quantified, **Figure 6C**, where Leu21 particle production is only slightly reduced, Val14 is reduced around 3-fold, Leu17 around 20-fold and in the double substitution no particles are detectable in the cell supernatant. Where particles were released they were tested for infectivity relative to *wt*, **Figure 6D**. Although the Val14 mutant showed greater defects in viral Env and Gag incorporation the viruses were only around 6-fold reduced in infectivity whereas viruses with the Leu21 substitution showed around a 300 fold reduction in infectivity and no infectivity (>100,00-fold reduced) was detectable for the Leu17 mutant. Taking these data together it is apparent that the Leu17 mutation has the least effect on *in vitro* Env binding but causes very large defects in PFV virion production with little, if any, incorporation of viral proteins into particles. The Leu21 substitution weakens the *in vitro* Gag-Env interaction more, has little effect on particle production but the resulting viruses are poorly infectious and viruses with the Val14 substitution display intermediate effects having both reduced particle production and reduced infectivity.

### Restriction of PFV and SFV_mac_ by Trim5α

**Figure 7:**
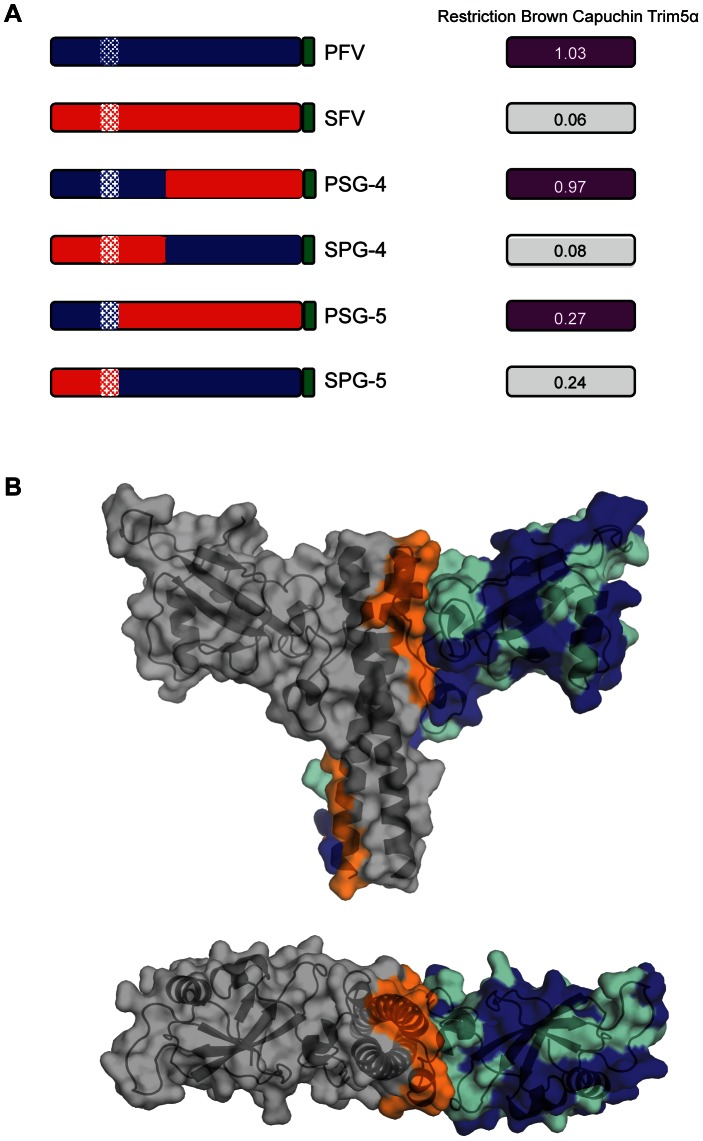
Restriction of foamy viruses. (**A**) Schematic bar representations of Gag from PFV (blue) and SFV_mac_ (red) along with chimeric PSG-4 (amino acids 1–311 PFV + 302–647 SFV), SPG-4 (amino acids 1–301 SFV + 312–648 PFV), PSG-5 (amino acids 1–195 PFV + 187–647 SFV) and SPG-5 (amino acids 1–186 SFV + 196–648 PFV) are shown on the left with C-terminal Gag P3 peptide coloured green and coiled coil regions hatched. Restriction of each virus by Brown Capuchin Trim5α is shown on the right. The values are the average from three independent experiments detailed in full in **Supplementary Figure S3**. (**B**) Mapping of the interfacial, conserved and non-conserved residues onto the PFV structure. The structure of PFV -Gag-Ntd is shown as a semi transparent surface surrounding a cartoon ribbon representation of the protein backbone. Residues that contribute to the dimer interface are shown in orange. Residues that are sequence conserved in SFV_mac_ and PFV are displayed in cyan and residues that are surface-exposed and non-conserved are displayed in blue.

Previous experiments have demonstrated that Gag from PFV and the closely related SFV_mac_ contain the target for Trim5α restriction. Moreover, PFV and SFV_mac_ display a differential susceptibility to restriction mediated by the B30.2 domain of Brown capuchin Trim5α (bc-T5α) that is effective only against SFV_mac_ and not PFV [36]. Based on sequence alignment, chimeras were prepared to more precisely map the target of Trim5α restriction in FV Gag. These included PSG-4 and SPG-4, that swap the N-terminal ∼300 residues between PFV and SFV_mac_ Gag and two further chimeras, one where the N-terminal 186 residues of SFV_mac_ Gag was replaced by the N-terminal 195 residues of PFV Gag (PSG-5) and a second where the N-terminal 195 residues of PFV Gag was replaced with the N-terminal 186 residues of SFV_mac_ Gag (SPG-5). The results of bc-T5α restriction assays of parent and chimeric PFV and SFV_mac_ viruses are summarised in **Figure 7A**, and detailed in **Supplementary Figure S3**. These data confirm that PFV is resistant to bc-T5α restriction and that SFV_mac_ is susceptible, and that sensitivity maps the N-terminal 300 amino acids of Gag, **Figure 7A** (PSG-4 and SPG-4). More importantly, these data also reveal that transfer of the N-terminal 186 residues of SFV_mac_ to PFV (SPG-5) now renders the virus susceptible to restriction by bc-T5α. Conversely, transfer of N-terminal 195 residues of PFV to SFV_mac_ (PSG-5) results in reduced sensitivity to bc-T5α restriction demonstrating that at least one determinant of restriction in primate FVs is contained within the Gag-NtD.

Since NtD s of PFV and SFV_mac_ Gag share a high degree of sequence similarity, the conserved residues along with those involved in the dimer interface were mapped onto the PFV-Gag-NtD structure, **Figure 7B**. Examination of this combined pattern of sequence conservation and surface accessibility reveals a large patch of surface exposed non-conserved residues on the upper surface of the molecule spanning from the β2–β3 loop across the outer surface of α2 and into the α2–α3 loop. The distribution of non-conserved residues over the top surface of the molecule is reminiscent of the distribution of residues that constitute the restriction factor binding sites in the N-terminal domain of the capsid of conventional retroviruses [55]. This suggests that the mode of foamy virus restriction by Trim5α is likely to be the same as in orthoretroviruses. In order to test this notion, mutations were introduced into the RING, B-Box and coiled coil domains of bc-T5α and the restriction of PFV and SFV_mac_ by these impaired factors assayed. These data, summarised in **Table 3** and detailed in **Supplementary Figure S4**, show that disruption of the individual RING and B-Box domains or deletion of the coiled-coil region completely abolishes bc-T5α restriction of SFV_mac_ and does not alter PFV susceptibility. Taken together with data demonstrating that the B30.2 domain of Trim5α mediates the Gag specificity of restriction [36] this demonstrates that FV restriction is reliant on the same functional regions required for orthoretrovirus restriction and likely occurs by the same mechanism.

**Table 3:** Brown Capuchin Trim5α restriction of PFV and SFV.

Mutant	PFV	SFV
aC15A/C18A	*1.05±0.03	0.94±0.04
bC95A/H98A	1.03±0.04	1.26±0.02
bW115E	1.01±0.02	1.24±0.06
bE118K/R119K	1.04±0.01	1.12±0.06
cΔ130–231	1.06±0.04	1.14±0.05
Trim5α *wt*	1.03±0.04	0.08±0.02

* Values are the average from three independent experiments detailed, **Supplementary Figure S4**.

a RING domain,

b B-box 2 domain,

c Coiled-coil domain.

## Discussion

### The Foamyvirus Gag-NtD is a unique structure

Based upon both the functional similarities and positioning within PFV Gag it might be expected that the Gag-NtD would display a strong structural similarity with MA of orthoretroviruses. However, following extensive searching of the Protein Database (PDB) no such similarity was apparent and in fact no structures related to PFV-Gag-NtD were found at all. Like FV-Gag-NtD, the orthoretroviral MA protein is required for targeting Gag to the membrane and for viral budding. This is accomplished through a combination of a highly basic region (HBR) and in some subfamilies a myristoyl group located at the N-terminus of MA [56], [57], [58]. However, although the MA functional properties are conserved, neither of these motifs is present in the PFV-Gag-NtD. Further, the structure of MA is highly conserved amongst retroviruses, consisting of a four α-helix globular core and an associated fifth helix [59], [60], [61], [62], [63], [64], [65]. By comparison, our data reveals the PFV-Gag-NtD to be entirely unrelated comprising a mixed α/β protein with head and stalk domains.

The dimeric organisation of FV-Gag-NtD is also not a conserved feature of orthoretroviral MAs. In HIV, myristoyl-MA promotes assembly and budding directly at the plasma membrane (PM) [56] and although it is unclear what the MA oligomerisation state is within immature and mature virions trimeric assemblies have been reported in vitro [61], [63]. In the betaretroviruses that like FVs assemble intracellularly at the pericentriolar region [32], [33], only weak self-association of MA has been demonstrated [66]. By contrast, in the delta-retrovirus HTLV-1 the presence of stable disulphide linked dimers of Gag and MA in both immature particles and mature virions has been observed [67]. Thus, although FV-Gag-NtD and orthoretroviral MA have membrane-targeting roles in the late part of the viral life cycle, the differences in structure and organisation suggests the existence of different evolutionary pathways.

Evidence for this notion also comes from sequence comparisons of the predicted Gag protein from FVs ranging from primate to sloth revealing they all share the same motifs and that they are unrelated to orthoretroviral Gag [11]. This implies there is one evolutionary pathway for the FVs with a single Gag protein and another for the orthoretroviruses in which the Gag precursor protein undergoes significant processing. Moreover, based on the observation of endogenous foamy virus in coelacanths, this divergence occurred more than 400 million years ago [13].

Foamy virus replication also has similarities with that of hepadnaviruses, including reverse transcription in the producer cell and an infectious DNA genome in the virion [24], [25]. As there is no apparent structural homology with orthoretroviral Gag one possibility is that FV Gag may be related to a hepadnavirus structural protein. Inspection of capsid protein of hepadnavirus B (Hep-B) [68] reveals that Hep-B CA is an all-helical protein with a prominent 4-helix bundle making up the interface between CA dimers. This arrangement is reminiscent of the coiled-coil dimer interface of the PFV-Gag, However, in Hep-B the 4-helix bundle forms “spikes” that protrude from the exterior of the capsid shell. Given the arrangement of FV Gag with the N-terminal MA layer found at the greatest radius and the more C-terminal regions of Gag projecting to the virion interior [29] it seems unlikely that FV Gag is related to hepadnavirus CA. This further supports the notion that FV Gag-NtD is the product of convergent evolution that has driven the formation of a unique structure with properties of orthoretroviral MA and CA.

### Location of the CTRS

**Figure 8:**
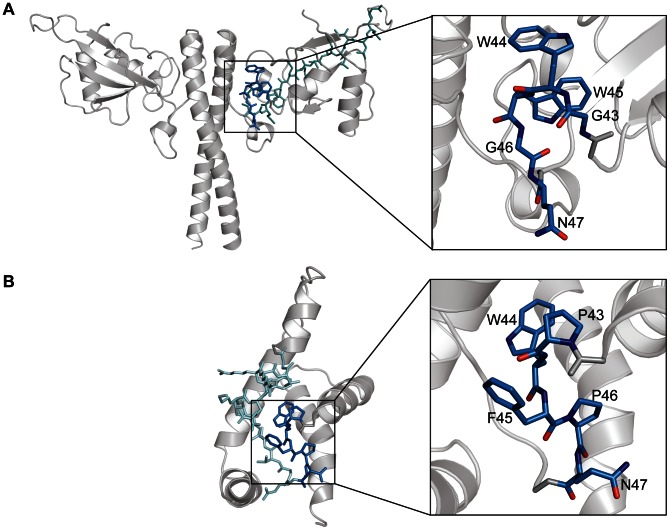
Location of CTRSs in PFV-Gag-Ntd and MPMV MA. (**A**) PFV-Gag-NtD and (**B**) MPMV MA are shown in cartoon representation. Residues that make up the proposed CTRS are shown in stick representation. The conserved di-aromatic motifs are highlighted in dark blue and shown in close up, in the right-hand panels.

The cytoplasmic targeting and retention signal (CTRS) found in the MA of betaretroviruses and in the Gag-NtD of FVs, promotes assembly in the pericentriolar region of the cell [32], [33], [34]. The consensus sequence in betaretroviruses spans residues Pro43 to Gly60 in MA of the archetypal betaretrovirus Mason-Pfizer monkey virus (MPMV) [69], [70]. Within this sequence the majority of residues, Pro43 to Ile53, constitute the loop that links helix α2 to helix α3 of MA whilst the remainder make up the first two turns of α3 [65]. In FVs the proposed CTRS constitutes residues 43 to 60 of the PFV-Gag-NtD [34] where residues Leu40 to Arg50 form the loop that links β1 to β2 and the remainder make up the β2 strand. Although the betaretroviral and FV CTRSs appear largely dissimilar, one common feature of both is a double aromatic motif G_43_WWGQ_47_ in PFV and P_43_WFPQ_47_ in MPMV. In both cases the sequences are located in the loop regions of the CTRS and comprise a structural motif consisting of a tight turn and a surface exposed aromatic and glutamine side chain, **Figure 8**. In MPMV, mutation of the CTRS causes Gag to traffic as a monomer to the plasma membrane where assembly and production of infectious virus still occurs [70]. By comparison, absence of a functional CTRS in FVs completely abrogates assembly and whilst addition of a myristoylation signal to PFV facilitates Gag trafficking to the plasma membrane, infectious particles are not produced [54], [71]. The severest effects on capsid formation and particle production were observed when alanine substitution mutations were introduced at Trp45 or Arg50 in the CTRS of PFV [53]. However, examination of the Gag-NtD structure now reveals that although Trp45 and Arg50 are part of the CTRS both are actually deeply buried in the core of the head domain. Arg50 also forms a number of important hydrogen bonds with neighbouring residues stabilising the interaction of the head domain with helix α5 immediately preceding the coiled-coil. Therefore, in these instances the severe mutational effects associated with alanine substitution can be likely attributed to destabilisation and/or misfolding of the Gag-NtD. However, mutation of the surface exposed Trp44 in the double aromatic motif does allow particle assembly but with a large reduction in both particle export and infectivity (∼10^5^ fold) [53]. In this case, given the exposure of the Trp44 sidechain, **Figure 8**, the lack of particle egress might be attributed directly to loss of a di-hydrophobic motif dependent CTRS function causing mislocalisation or incorrect trafficking of assembled virions.

### Env-Gag interactions

FV egress requires interaction between the Gag and Env proteins to ensure correct membrane trafficking and viral budding. It seems likely that FV Gag becomes associated with Env through interaction with Env leader peptide (Env-LP) displayed on the cytosolic side of the ER and Trans-Golgi network (TGN) after core assembly at the pericentriolar region [31], [33]. Env then directs the intracellular transport of the assembled particles to enable mature viruses to bud at the PM or sometimes into intracellular vacuoles. This interaction guarantees Env incorporation into virions and the loss of either interacting domain (Gag-NtD or Env-LP) results in the intracellular stranding of assembled FV capsids [30], [52].

Mutations in the Env binding site of PFV-Gag-NtD have been shown to affect viral assembly, egress and infectivity. Of note is Leu17 that when substituted by serine results in loss of virus production, **Figure 6A**. However, our *in vitro* binding data, **Figure 5**, reveal only modest reductions in affinity (2–5 fold) when single serine substitutions are introduced into the Env binding site suggesting that the Leu17 to serine mutation may have effects prior to Gag-Env association. This notion is further supported by the fact that the mutant displays a phenotype similar to that of the Trp45 and Arg50 alanine mutations that disrupt the CTRS [53]. Examination of virion production and Env incorporation in Val14 and Leu21 serine substitution mutants reveals reduced levels in Val14 serine mutant but near wild type amounts in Leu21 particles. The small effects on virus production observed with the Leu21 and Val14 mutations also correlate well with the modest reductions in K_A_ observed with the single-site mutations. This likely reflects the situation that recruitment of Env by a preassembled FV core rather than by Gag monomers is subject to the avidity effects of having many Gag binding sites arrayed on the core surface. Therefore, even under conditions of reduced binding the cores can still recruit enough Env to bud efficiently. However, whilst the effects on particle number and Gag-Env interaction are small, the Val14 and Leu21 serine mutations result in reduced infectivity, similar to when Leu17 is replaced by alanine [53], suggesting that disruption of the Gag-Env interaction may also be detrimental for post-entry events in the target cells.

In the structure, residues 7–16 of the Env leader peptide comprise the amphipathic α-helix bound in the Env binding site of the Gag-NtD and residues 1–6 provide intramolecular hydrogen bonding that stabilises the helical conformation. The affinity of the interaction, 1.5×10^5^ M^−1^, is comparable with the value of 0.65×10^5^ M^−1^ reported for the interaction of residues 1–30 of the FFV Env leader peptide with the equivalent Gag-NtD [29]. Therefore, the hydrophobic interface observed in the structure likely represents the complete interaction between the leader peptide and FV Gag. The apolar character of the Env binding site is largely conserved among primate FVs although there is significant variation in the primary sequences of the α1 helix, **Figure 1C**. By contrast, the sequences of the N-terminal 13 residues of the Env leader peptide are largely invariant giving rise to the conserved motif [M-A-P-P-M-(T/S/N)-L-(E/Q)-Q-W-Φ-Φ-W] where Φ denotes a residue with a hydrophobic side chain. Our binding data show that removal of the first 4 residues (MAPP) along with Ala19 and His20, not visible in the crystal structure, results in a significant reduction in Gag-Env binding, **Figure 3**. It has also been demonstrated previously that the N-terminal four residues as well as the conserved tryptophan residues Trp10^ENV^ and Trp13^ENV^, are essential for PFV egress [31]. Moreover, mutation of the equivalent conserved tryptophans in FFV greatly reduces the Gag-Env interaction *in vitro* [29]. The necessity for the N-terminal five residues is now apparent from the Gag-Env complex structure as many of the residues in the N-terminal extended region make polar contacts with Gag but also make intramolecular interactions with the Env helix to stabilise the conformation that binds to the Gag. The importance of the tryptophans is also apparent as they form part of the hydrophobic interface with Gag. Given the degree of conservation in the N-terminal of Env it is likely that this mode of interaction is a common feature of the Env-LP interaction with the Gag-NtD in other FVs.

### Capsid structure and restriction

In orthoretroviruses, the viral core is enclosed by a hexameric lattice of CA assembled through combined homotypic and heterotypic interactions mediated by the amino-terminal (CA-NtD) and carboxy-terminal (CA-CtD) domains of CA [39], [51], [72], [73]. In FVs, the structural organisation of the core is less characterised but two regions of FV-Gag required for assembly have been identified. Reminiscent of orthoretroviral CA-NtD and CA-CtD, the first corresponds to the Gag-NtD coiled-coil dimer defined in our structural studies [74] (**Figure 1**) and the other found in the central region of FV-Gag (Gag-CtD) includes a conserved YXXLGL assembly motif [75]. In all likelihood the interior structural organisation of the FV virion is also formed by combinatorial heterotypic and homotypic protein-protein interactions mediated by these assembly domains, although the requirement for other regions of Gag, not yet identified, cannot be excluded. A further functional similarity of FV Gag-NtD and orthoretroviral CA-NtD is that both appear to be the target of Trim5α, mediated restriction **Figure 7**, [36], [37] and in orthoretroviruses, it is proposed that underlying hexagonal pattern of the assembled CA is recognised by a complementary hexagonal assembly of Trim5α in order to initiate the restriction process [76], [77]. Presently, the overall arrangement of the Gag protein in an assembled FV is unknown but since the same species dependent Trim5α restriction of PFV and other FVs is apparent [35], [36] the requirement for a lattice structure that arrays FV-Gag-NtD on the exterior of the FV core might also be expected. One possibility is that FV-Gag-NtD dimerisation combined with FV-Gag-CtD interactions generates a higher-order hexagonal Gag assembly targeted by Trim5α factors. However, given the obligate nature of the FV Gag-NtD dimer together with its organisation, dimensions and lack of structural homology with orthoretroviral CA it is difficult to envisage how a hexagonal assembly of equivalent spacing to that of the orthoretroviruses might be present in the FV particle. These observations raise the question of whether Trim5α might target other regular, or even irregular, molecular arrangements in addition to the hexagonal assemblies. Current models rely on a rather rigid overlapping of the orthoretroviral CA and Trim5α supramolecular assemblies. The inclusion of FVs in the cadre of Trim5α targets suggests there is potential flexibility in the pattern recognition receptor activity of Trim5α. Determining how this is accomplished awaits further structural and microscopic studies of the FV virion.

## Materials and Methods

### Cells and viruses

Human HT1080 [78] and 293T [79] cells were maintained in Dulbecco modified Eagle medium supplemented with 10% foetal calf serum and 1% penicillin and streptomycin. Restriction factors were delivered into cells using Moloney MLV (MoMLV)-based vectors produced by transfection of 293T cells. MoMLV-based delivery vectors were made by co-transfection of VSVG, pHIT60, and pLgatewayIRESEYFP containing the restriction gene. FVs were produced by a four-plasmid PFV vector co-transfection system [80], [81] in which pciSFV-1env (providing Env), pcziPol PFV vector (providing Pol), pMD9 (a minimal vector genome with an EGFP marker gene), and a Gag-expressing construct were co-transfected. FV vector supernatants were harvested 48 h post-transfection, aliquoted, and stored at −80°C until further use. Subsequently, individual vector supernatant aliquots were pre-titrated on HT1080 cells using the EGFP marker gene and flow cytometric analysis. For the two-colour restriction assay described below, FV vector supernatants were then used at dilutions that resulted in 3 to 40% EGFP-positive HT1080 cells.

A chimeric TRIM5α with the RBCC domain of human TRIM5α and the PRYSPRY domain of brown capuchin, referred to here as capuchin TRIM5α because the PRYSPRY domain determines restriction specificity, has been described previously [82]. A series of mutants of this factor in RING (C15A/C18A), B-box 2 (C95A/H98A, W115E and E118K/R119K) and coiled-coil (delta 130–231) were prepared by site directed mutagenesis.

Preparation of the PFV and SFV packaging plasmids, PFV pcziGag4 (PGWT) and SFV_mac_ pcziSG (SGWT) respectively, as well as chimeric PFV/SFV Gag packaging constructs, PSG-4 and SPG-4, has been described previously [36]. The novel chimeric constructs PSG-5 and SPG-5 were generated by recombinant overlap PCR starting with PGWT and SGWT. PSG-5 contains amino acids 1–195 of PFV and 187–647 of SFV while SPG-5 encodes amino acids 1–186 of SFV and 196–648 of PFV.

PFV Gag point mutants were generated in context of a the original PFV Gag packaging construct pcziGag4 [80] (L17S/L21S), or a C-terminally HA-tagged variant thereof, pcziPG CLHH (V14S, L17S, L21S).

### Restriction assays

Restriction was determined by our previously described two-colour fluorescence activated cell sorter (FACS) assay [83]. Briefly, HT1080 cells were transduced with the MLV-based pLgatewayIRESEYFP retroviral vector carrying the restriction gene and an EYFP marker gene 2 days prior to challenging with FVs carrying the EGFP marker. The percentage of YFP positive cells (i.e. restriction factor-positive cells) that were EGFP positive (i.e. FV infected) was then determined by FACS. This was compared to the percentage of FV-infected cells (EGFP positive) in cells that did not express the restriction factor (EYFP negative). A ratio that was less than 0.3 was taken to represent restriction, while a ratio greater than 0.7 indicated the absence of restriction.

### Particle release and infectivity assay

Cell culture supernatants containing recombinant viral particles were generated as described previously [84]. Briefly, 293T cells were co-transfected in 10 cm dishes with a Gag expression plasmid (pcziGag4 or PG mutants thereof, as indicated), Env (pcoPE), Pol (pcoPP), and the transfer vector (puc2MD9) at a ratio of 16∶1∶2∶16 using Polyethyleneimine (PEI) reagent and 16 µg DNA total. At 48 h post transfection (p.t.) cell-free viral vector supernatant was harvested using 0.45 µm sterile filters.

For transduction efficiency analysis 2×10^4^ HT1080 cells were plated in 12-well plates 24 h before infection. The target cells were incubated with 1 ml of plain cell-free viral supernatant or serial dilutions thereof for four to six hours. Determination of the percentage of eGFP-expressing cells was performed 72 h after infection by flow cytometry analysis and used for titre determination as previously described [85]. All transduction experiments were repeated at least three times. To compare the infectivity in repetitive experiments the titre obtained for wild type supernatants in individual experiments was set to an arbitrary value of 100%. The other values were then normalized as percentage of the wild type value.

Viral protein expression in transfected cells and particle-associated protein composition was examined by Western blot analysis. Preparation of cell lysates from one transfected 10-cm cell culture dish was performed by incubation with 0.6 ml lysis buffer for 20 min at 4°C followed by centrifugation through a QIAshredder (Qiagen). All protein samples were mixed with equal volumes of 2×PPPC (100 mM Tris-HCl; pH 6.8, 24% glycerol, 8% SDS, 0.2% Bromophenol blue, 2% ß-mercaptoethanol) prior to separation by SDS-PAGE using 7.5% polyacrylamide gels. Viral particles were concentrated from cell-free supernatant of transfected 293T cells by ultracentrifugation through a 20% sucrose cushion at 4°C and 25,000 rpm for 3 h in an SW32 rotor. The viral pellet was resuspended in phosphate-buffered saline (PBS). Immunoblotting using polyclonal antisera specific for PFV Gag [86] or PFV Env leader peptide [75] was performed as previously described [31]. The chemiluminescence signal was digitally recorded using a LAS3000 imager and quantified using ImageGauge in the linear-range of the sample signal intensities as described previously [87].

### Protein expression

The DNA sequences coding for PFV-Gag residues 1–179 (PFV-Gag-NtD) and FFV residues 1–154 (FFV-Gag-NtD) were amplified by PCR from template plasmids pcziGag4 and pcDWF003 containing the PFV and FFV Gag genes respectively. PCR products were inserted into a pET47b expression vector (Novagen) using ligation independent cloning in order to produce N-terminal His-tag fusions with 3C protease cleavage sites. The correct sequence of expression constructs was verified by automated DNA sequencing (Beckman Coulter Genomics). His-tagged PFV- and FFV-Gag-NtD were expressed in the *E. coli* strain Rosetta 2 (DE3) and purified using Ni-NTA affinity (Qiagen) and size exclusion chromatography on Superdex 200 (GE healthcare). Selenium was incorporated into PFV-Gag-NtD by replacement of methionine with seleno-methionine in defined culture medium and by inhibition of methionine biosynthesis just prior to IPTG induction [88]. Verification of the processed N-terminal methionine, correct molecular mass and degree of selenium incorporation was obtained by electrospray ionisation mass-spectrometry. Peptides comprising residues 1–20 and 5–18 from the PFV-Env leader region were purchased HPLC purified from Pepceuticals Ltd.

### SEC-MALLS

Size exclusion chromatography coupled multi-angle laser light scattering (SEC-MALLS) was used to determine the molar mass of FFV- and PFV-Gag-Ntd. Samples ranging from 1.5 to 12.0 mgml^−1^ were applied in a volume of 100 µl to a Superdex 200 10/300 GL column equilibrated in 20 mM Tris-HCl, 150 mM NaCl and 0.5 mM TCEP, pH 8.0, at a flow rate of 0.5 ml/min. The scattered light intensity and the protein concentration of the column eluate were recorded using a DAWN-HELEOS laser photometer and OPTILAB-rEX differential refractometer respectively. The weight-averaged molecular mass of material contained in chromatographic peaks was determined from the combined data from both detectors using the ASTRA software version 6.0.3 (Wyatt Technology Corp., Santa Barbara, CA, USA).

### Analytical ultracentrifugation

Sedimentation velocity experiments were performed in a Beckman Optima Xl-I analytical ultracentrifuge using conventional aluminium double sector centrepieces and sapphire windows. Solvent density and the protein partial specific volumes were determined as described [89]. Prior to centrifugation, samples were prepared by exhaustive dialysis against the buffer blank solution, 20 mM Tris-HCl, 150 mM NaCl and 0.5 mM TCEP, pH 7.5. Centrifugation was performed at 50,000 rpm and 293 K in an An50-Ti rotor. Interference data were acquired at time intervals of 180 sec at varying sample concentration (0.5–2.5 mg/ml). Data recorded from moving boundaries was analysed using both a discrete species model and in terms of the size distribution functions C(S) using the program SEDFIT [90], [91], [92]. For analysis of Env peptide binding, sedimentation velocity experiments were conducted in 3 mm pathlength centrepieces using equimolar mixtures (75 µM) of PFV-Gag-NtD and Env peptides. In these experiments, radial absorbance scans at 280 nm were also recorded along with the interference data.

Sedimentation equilibrium experiments were performed in a Beckman Optima XL-I analytical ultracentrifuge using charcoal filled Epon six-channel centrepieces in an An-50 Ti rotor. Prior to centrifugation, samples were dialyzed exhaustively against the buffer blank, 20 mM Tris-HCl, pH 7.5, 150 mM NaCl, 0.5 mM TCEP. After centrifugation for 18 h, interference data was collected 2-h intervals until no further change in the profiles was observed. The rotor speed was then increased and the procedure repeated. Data were collected on samples of different concentrations of FFV- and PFV-Gag-NtD (14–100 µM) at three speeds and the program SEDPHAT [93], [94] was used to determine weight-averaged molecular masses by nonlinear fitting of individual multi-speed equilibrium profiles (A versus r) to a single-species ideal solution model. Inspection of these data revealed that the molecular masses showed no significant concentration dependency and so global fitting incorporating the data from multiple speeds and multiple sample concentrations was applied to extract a final weight-averaged molecular mass.

### Quantitation of sedimentation binding data

Data were analysed using a general binding expression, Eq. 1. This expression relates the association constant K_a_ to the fraction of bound peptide θ (θ = [PL]/[Lt]) in terms of the total concentrations of peptide [Lt] and protein [Pt] and is a modification of the formulae employed in [95], [96].
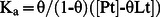
(1)In sedimentation velocity experiments θ was determined from the integrated absorbance of the 3S species in the C(S) function that best fits the sedimentation data. As equimolar ratios of peptide and protein were employed ([Lt] = [Pt]) Eq. 1 can be simplified and equilibrium association constants determined from Eq. 2.
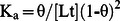
(2)


### Isothermal titration calorimetry

ITC was carried out using an ITC-200 calorimeter (MicroCal). Briefly, PFV-Gag-NtD was prepared by were dialysis against 25 mM Na-phosphate pH 6.55, 100 mM NaCl, 0.5 mM TCEP. A typical experiment involved 20 injections of 1 mM Env peptide in the injection syringe into 50 µM PFV-Gag-NtD in the sample cell. Data was analysed using the Origin-based software provided by the manufacturers.

### Protein crystallisation and structure determination

PFV-Gag-NtD was crystallised using hanging drop vapour diffusion. Typically, A 10 mg/ml solution of PFV-Gag-NtD in 150 mM NaCl, 5% glycerol, 10 mM Tris-HCl, pH 8.0 was mixed with an equal volume of crystallisation solution containing 16% PEG 6000 (w/v), 12% ethylene glycol, 0.03 M MgCl_2_ hexahydrate and suspended over a reservoir of the crystallisation solution. Crystals appeared within 14 days and were transferred into fresh crystallisation solution supplemented with 20% glycerol and flash-frozen in liquid nitrogen. The crystals belong to the space group P2_1_ with one copy of the PFV dimer in the asymmetric unit. Seleno-methionine derived protein was crystallized under the same conditions. Crystals of the PFV-Gag-NtD-Env complex were also grown by vapour diffusion by mixing 500 µM 1∶1 complex in 150 mM NaCl, 5% glycerol, 10 mM Tris-HCl, pH 8.0 with an equal volume a crystallisation solution containing 10% PEG 4000 (w/v), 20% glycerol, 0.03 M MgCl_2_, 0.03 M CaCl_2_, 0.1 M Tris-Bicine pH 8.5. Crystals appeared within 2 days and were harvested into fresh crystallisation solution supplemented with 20% glycerol and flash-frozen in liquid nitrogen prior to data collection. Crystals of the complex also belong to the space group P2_1_ but with two copies of the PFV dimer-peptide complex in the asymmetric unit. The structure of PFV-Gag-NtD was solved by single wavelength anomalous diffraction (SAD) using a dataset recorded at 0.9791 Å at 100 K on beamline I03 at the Diamond Light Source (Didcot, UK) using crystals of the seleno-methionine substituted protein. Data was processed using the HKL program package [97] and 13 selenium atoms were located by SAD methods in PHENIX [98]. Further density modification in PHENIX resulted in a figure of merit of 0.79 and a map of sufficient quality for a near complete model to be built using Arp/Warp [99]. The model was completed by iterative rounds of refinement and model building in PHENIX and COOT [100]. TLS groups were included in final round of refinement as determined by TLSMD [101]. The structure was refined to a final R_work_/R_free_ of 17.2/23.0 respectively and has good geometry with 98.8% of residues in the preferred region of the Ramachandran plot, only 1.2% in the additionally allowed region and no outliers. Details of crystal parameters and data refinement statistics are presented in **Table 1**. Data for the PFV-Gag-NtD-Env complex was collected at 100 K on beamline I03 and processed and scaled in space group P2_1_ using XDS/XSCALE [102]. The structure was solved by molecular replacement using Phaser [103] with the Gag-NtD dimer used as a search model to locate the two copies of the complex in the asymmetric unit. The model was completed by iterative rounds of TLS based refinement and model building using Refmac5 [104] and COOT. TLS groups were defined using TLSMD. The structure was refined to a final R_work_/R_free_ of 22.6/27.1 in which 98.8% of residues lie within preferred regions of the Ramachandran plot and the remaining 1.2% residues lie within the additionally allowed region. The crystal and refinement parameters are given in **Table 1**. The coordinates and structure factors of PFV-Gag-NtD and PFV-Gag-NtD-Env complex have been deposited in the Protein Data Bank under accession numbers 4JNH and 4JMR respectively.

## Supporting Information

Figure S1 Structural alignment of free and bound PFV-Gag-NtD. (**A**) Structurally aligned free and bound PFV-Gag-NtD are shown in cartoon representation and the bound Env peptides as cylinders. Regions that align well are shaded grey in both structures. Loop regions were significant deviations occur, the α1-β1 and β3–β4 loops, are coloured blue (free) and green (bound). (**B**) Close up view of the β3–β4 loop shown in stick representation. Free and bound are coloured as in **A** and residues labelled. (**C**) Close up view of the α1-β1 loop and the Env binding site in stick representation. Free and bound are coloured as in **A** and residues around Pro30 were the largest deviations occur are labelled.
(TIF)

Figure S2 Sedimentation velocity analysis of Gag-Env interface mutants. C(S) functions that best fit sedimentation velocity profiles for Env binding experiments from (**A**) V18S, (**B**) L21S, (**C**) L21N, (**D**) V14S/V18SS, (**E**) V18S/L21S and (**F**) V14S/V18S/L21S mutants. The C(S) function from 75 µM Gag NTD (black) and from 75 µM equimolar mixtures of Gag NtDs with Env_1–20_ (red) are shown in each panel.
(TIF)

Figure S3 Restriction of chimeric PFV and SFV_mac_. The table shows the degree of restriction of PFV and SFV_mac_ along with chimeric PGS-4, SPG-4, PSG-5 and SPG-5. Values are the ratio of the percentage of infected restriction factor-positive cells to the percentage of infected cells not expressing the restriction. A lower than 0.3 was taken to represent restriction, while a ratio greater than 0.7 indicated the absence of restriction. The data shown are the means and standard deviations from triplicate independent experiments.
(TIF)

Figure S4 Restriction of PFV and SFV_mac_ by Brown Capuchin Trim5α. The table shows the results of restriction assays were mutations have been introduced into a Human-Brown Capuchin Trim5α hybrid comprising the RING, B-box and coiled coil domains of human Trim5α and the B30.2 domain from Brown Capuchin Trim5α. Values are the ratio of the percentage of infected restriction factor-positive cells to the percentage of infected cells not expressing the restriction. A lower than 0.3 was taken to represent restriction, while a ratio greater than 0.7 indicated the absence of restriction. Each experiment has been repeated 3–4 times, errors are standard deviations of triplicates in each independent experiment. Mutations in the RING, B-Box and coiled coil all result in a complete loss of restriction activity against SFV_mac_ and no gain of restriction activity against PFV.
(TIF)
