# Supplementary figures and images for: A Unique Spumavirus Gag N-terminal Domain with Functional Properties of Orthoretroviral Matrix and Capsid
Source: PLoS Pathog. 2013 May 9;9(5):e1003376. doi: 10.1371/journal.ppat.1003376 (PMC3649970; doi:10.1371/journal.ppat.1003376)

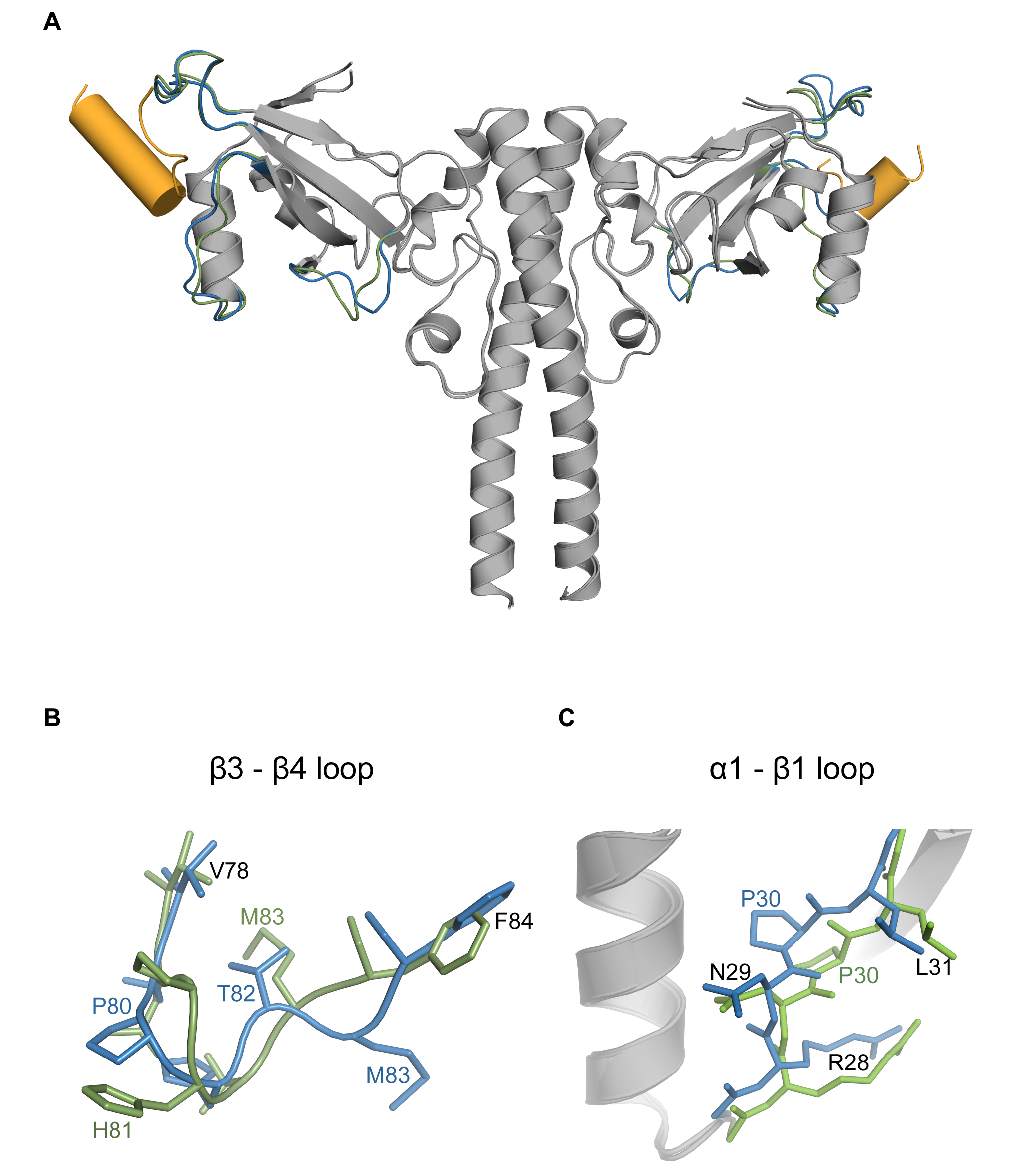

Supplement: Figure S1 — Structural alignment of free and bound PFV-Gag-NtD. (A) Structurally aligned free and bound PFV-Gag-NtD are shown in cartoon representation and the bound Env peptides as cylinders. Regions that align well are shaded grey in both structures. Loop regions were significant deviations occur, the α1-β1 and β3–β4 loops, are coloured blue (free) and green (bound). (B) Close up view of the β3–β4 loop shown in stick representation. Free and bound are coloured as in A and residues labelled. (C) Close up view of the α1-β1 loop and the Env binding site in stick representation. Free and bound are coloured as in A and residues around Pro30 were the largest deviations occur are labelled. (TIF) [file ppat.1003376.s001.tif]

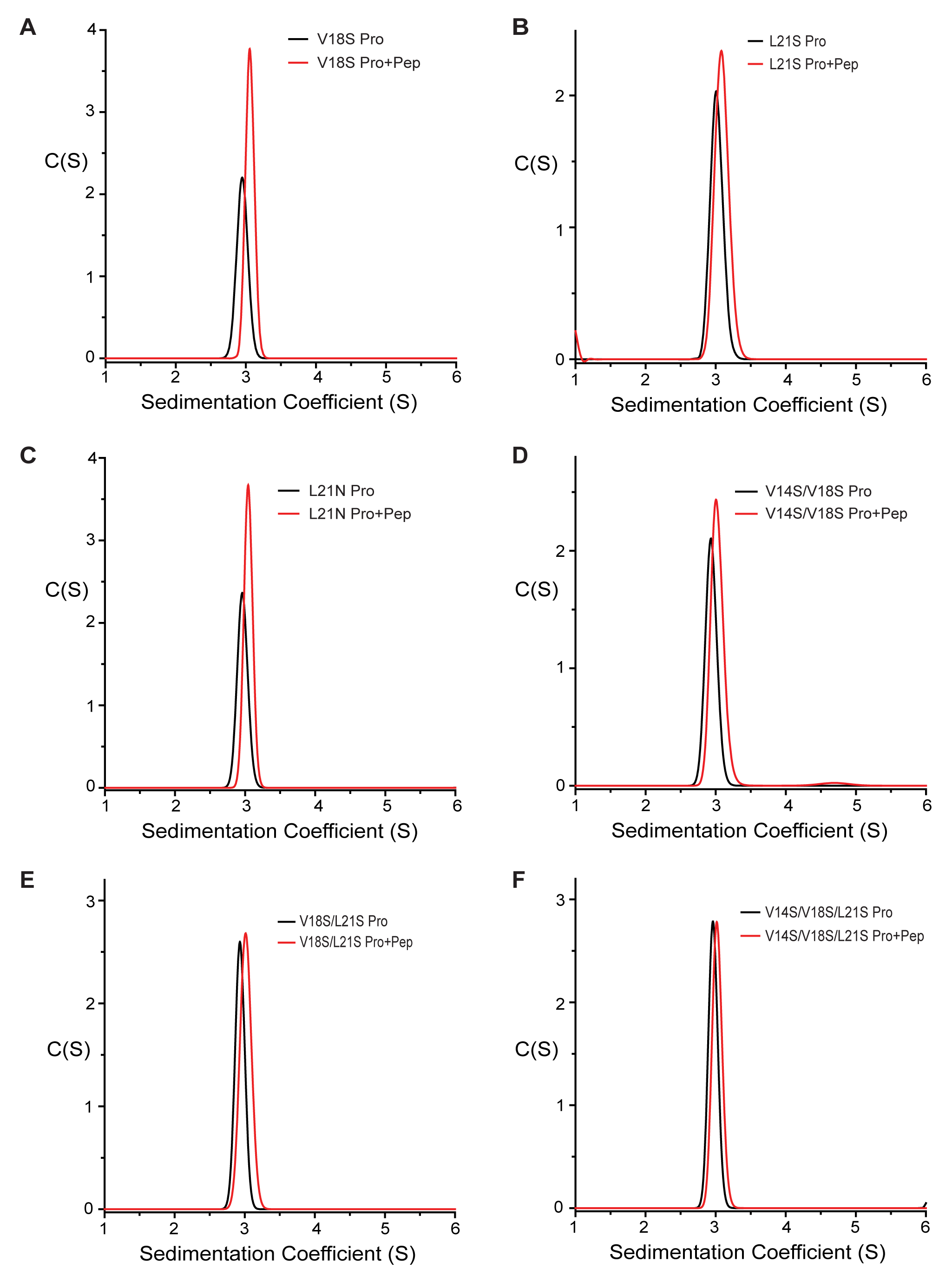

Supplement: Figure S2 — Sedimentation velocity analysis of Gag-Env interface mutants. C(S) functions that best fit sedimentation velocity profiles for Env binding experiments from (A) V18S, (B) L21S, (C) L21N, (D) V14S/V18SS, (E) V18S/L21S and (F) V14S/V18S/L21S mutants. The C(S) function from 75 µM Gag NTD (black) and from 75 µM equimolar mixtures of Gag NtDs with Env1–20 (red) are shown in each panel. (TIF) [file ppat.1003376.s002.tif]

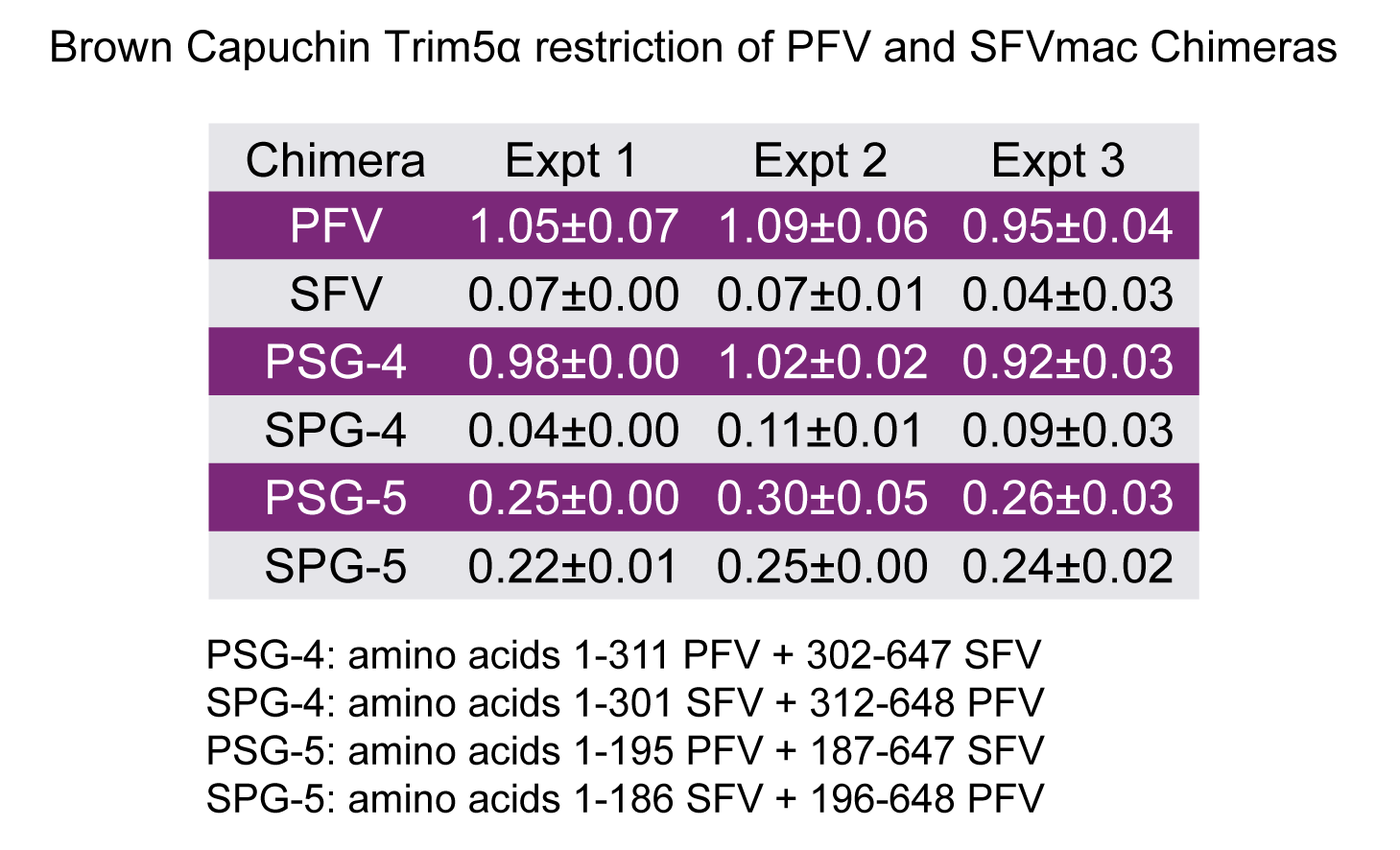

Supplement: Figure S3 — Restriction of chimeric PFV and SFVmac. The table shows the degree of restriction of PFV and SFVmac along with chimeric PGS-4, SPG-4, PSG-5 and SPG-5. Values are the ratio of the percentage of infected restriction factor-positive cells to the percentage of infected cells not expressing the restriction. A lower than 0.3 was taken to represent restriction, while a ratio greater than 0.7 indicated the absence of restriction. The data shown are the means and standard deviations from triplicate independent experiments. (TIF) [file ppat.1003376.s003.tif]

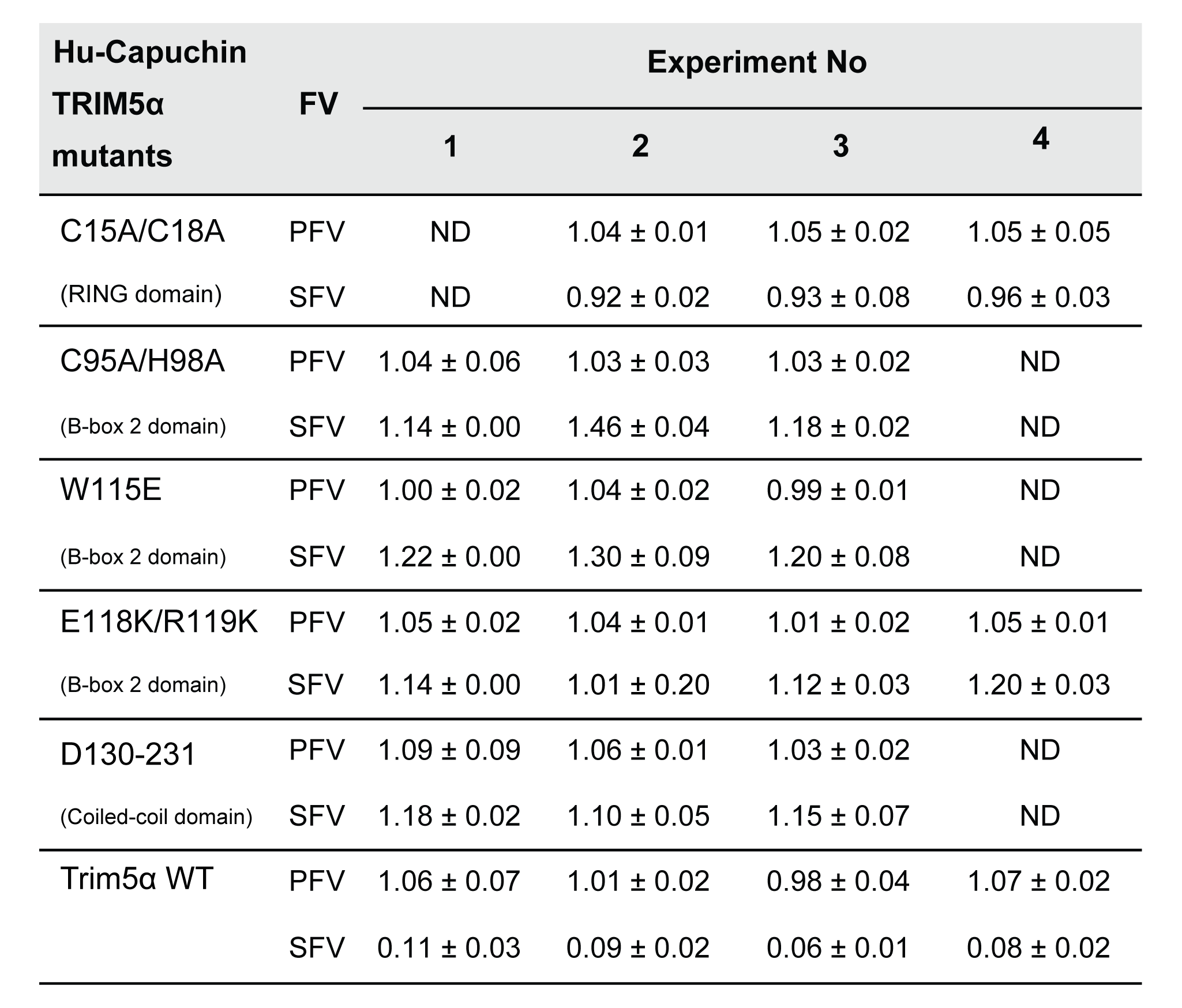

Supplement: Figure S4 — Restriction of PFV and SFVmac by Brown Capuchin Trim5α. The table shows the results of restriction assays were mutations have been introduced into a Human-Brown Capuchin Trim5α hybrid comprising the RING, B-box and coiled coil domains of human Trim5α and the B30.2 domain from Brown Capuchin Trim5α. Values are the ratio of the percentage of infected restriction factor-positive cells to the percentage of infected cells not expressing the restriction. A lower than 0.3 was taken to represent restriction, while a ratio greater than 0.7 indicated the absence of restriction. Each experiment has been repeated 3–4 times, errors are standard deviations of triplicates in each independent experiment. Mutations in the RING, B-Box and coiled coil all result in a complete loss of restriction activity against SFVmac and no gain of restriction activity against PFV. (TIF) [file ppat.1003376.s004.tif]
